# A New Assessment of Bicycle Helmets: The Brain Injury Mitigation Effects of New Technologies in Oblique Impacts

**DOI:** 10.1007/s10439-021-02785-0

**Published:** 2021-05-10

**Authors:** Fady Abayazid, Ke Ding, Karl Zimmerman, Helena Stigson, Mazdak Ghajari

**Affiliations:** 1 Dyson School of Design Engineering, Imperial College, London, UK; 2 Computational, Cognitive and Clinical Neuroimaging Laboratory, Department of Brain Sciences, Hammersmith Hospital, Imperial College London, London, UK; 3 Folksam Insurance Group, Stockholm, Sweden; 4 Vehicle Safety Division, Department of Applied Mechanics, Chalmers University of Technology, Gothenburg, Sweden; 5 Division of Insurance Medicine, Department of Clinical Neuroscience, Karolinska Institutet, 171 77 Stockholm, Sweden

**Keywords:** Traumatic brain injury, Helmets, Rotational motion, Oblique impacts, Standards

## Abstract

New helmet technologies have been developed to improve the mitigation of traumatic brain injury (TBI) in bicycle accidents. However, their effectiveness under oblique impacts, which produce more strains in the brain in comparison with vertical impacts adopted by helmet standards, is still unclear. Here we used a new method to assess the brain injury prevention effects of 27 bicycle helmets in oblique impacts, including helmets fitted with a friction-reducing layer (MIPS), a shearing pad (SPIN), a wavy cellular liner (WaveCel), an airbag helmet (Hövding) and a number of conventional helmets. We tested whether helmets fitted with the new technologies can provide better brain protection than conventional helmets. Each helmeted headform was dropped onto a 45° inclined anvil at 6.3 m/s at three locations, with each impact location producing a dominant head rotation about one anatomical axes of the head. A detailed computational model of TBI was used to determine strain distribution across the brain and in key anatomical regions, the corpus callosum and sulci. Our results show that, in comparison with conventional helmets, the majority of helmets incorporating new technologies significantly reduced peak rotational acceleration and velocity and maximal strain in corpus callosum and sulci. Only one helmet with MIPS significantly increased strain in the corpus collosum. The helmets fitted with MIPS and WaveCel were more effective in reducing strain in impacts producing sagittal rotations and a helmet fitted with SPIN in coronal rotations. The airbag helmet was effective in reducing brain strain in all impacts, however, peak rotational velocity and brain strain heavily depended on the analysis time. These results suggest that incorporating different impact locations in future oblique impact test methods and designing helmet technologies for the mitigation of head rotation in different planes are key to reducing brain injuries in bicycle accidents.

## Introduction

Cycling is the most popular mode of active mobility, with many environmental and health benefits.^25^,^38^,^39^ The number of cyclists are steadily increasing in Europe, United States and worldwide since 2009.^2^,^12^ For instance, the pedal cyclist traffic increased by 16% in Great Britain between 2009 and 2019.^33^ The recent COVID-19 pandemic has led to a large increase in cyclist traffic, which is likely to be permanent. The UK’s Secretary of State for Transport has reported “*We’ve seen around a 100% increase in weekday cycling. At weekends, that increase has been up to around 200% compared to pre-COVID-19 levels. We want to use this recovery to permanently change the way we travel with huge levels of investment.*”.^51^

However, cyclists are among the vulnerable road users. Their severe injury and fatality rate per passenger miles are several folds larger than car occupants and bus passengers.^41^ More cyclists were fatally injured in 2018 than in any year since 1990 in the U.S. according to the U.S. Department of Transportation.^12^ Notably, the head is the most common body part to be severely injured during an accident.^46^ For instance, an analysis of the STRADA (Swedish Traffic Accident Data Acquisition) database showed that 42% of injuries leading to severe impairment were blows to the head.^46^ Impacts to the head can lead to traumatic brain injury (TBI) with fatal and lifelong consequences and large economic costs.^3^ Hence, cyclists are often advised to wear helmets as helmets can play a key role in protecting their head and brain against impacts.^13^,^17^,^20^ Previous work has shown that 19% of helmeted cyclists suffered severe TBI compared to 48% of non-helmeted cyclists.^17^ This study, amongst others, shows that there are still opportunities to reduce TBI in helmeted cyclists through improving helmet design.

The functional design of bicycle helmets has been driven by standard test methods (e.g. EN1078), where helmets are assessed under vertical impacts and the linear motion of the headform is used to evaluate their protection effects.^19^ However, analysis of accident data shows that in vast majority of real-world head collisions, impacts to the head occur at an angle which produces large rotational motions. There is significant body of research that show rotational motion of the head is the key determinant of brain deformation and subsequent damage to the brain tissue.^27^,^30^,^42^,^50^ These studies have led to new proposals from Fédération Internationale de Motocyclisme (FIM) and European Committee for Standardization Working Group 11 (CEN/TC158/WG11) for helmet testing under oblique impacts and using injury criteria based on head rotation.^24^,^35^,^37^,^49^ However, the effects of current bicycle helmets, particularly those that incorporate new technologies to reduce head rotation, on mitigating brain injuries under oblique impacts are still unclear.

Limited studies have assessed the performance of bicycle helmets with new technologies dedicated to mitigating rotational head motion.^8^,^9^ These previous studies assessed the performance of helmets in a single impact location. In contrast, a significant body of research has shown that the location and direction of impact has a large effect on rotational kinematics of the head and brain deformation.^7^,^31^,^35^,^50^ In addition, helmets are likely to provide different levels of protection against impacts at different locations.^15^ Hence, it is important to assess the performance of helmets under oblique impacts with different directions and locations.

In this study, we evaluated brain protection effects of a range of commercially available helmets under different oblique impacts. We studied helmets with EPS liners (conventional), helmets fitted with the friction-reducing ‘multi-directional impact protection system’ (MIPS),^8^ helmets with a corrugated ‘wavy’ cellular liner (WaveCel),^8^ helmets fitted with shearing pads (SPIN) and the airbag helmet Hövding 3.0.^32^ We tested whether helmets fitted with these new technologies provide better or worse brain protection in oblique impacts than conventional helmets.

Previous computational studies have shown that head impacts can produce large mechanical strains in key brain regions; corpus callosum and sulci.^22^,^26^ The corpus callosum is the largest white matter tract, which connects two hemispheres and is a location typically associated with diffuse axonal injury after head impacts.^47^ Sulci is where the pathology of the neurodegenerative disease, chronic traumatic encephalopathy, in sporting collisions and white matter damage in survivors of single head impacts have been seen.^22^,^34^ Hence, in addition to using measures of brain injury based on head kinematics, we used a detailed finite element model of TBI to predict strain in the sulci and corpus callosum during oblique impacts.

## Methods

### Bicycle Helmets

**Figure 1:**
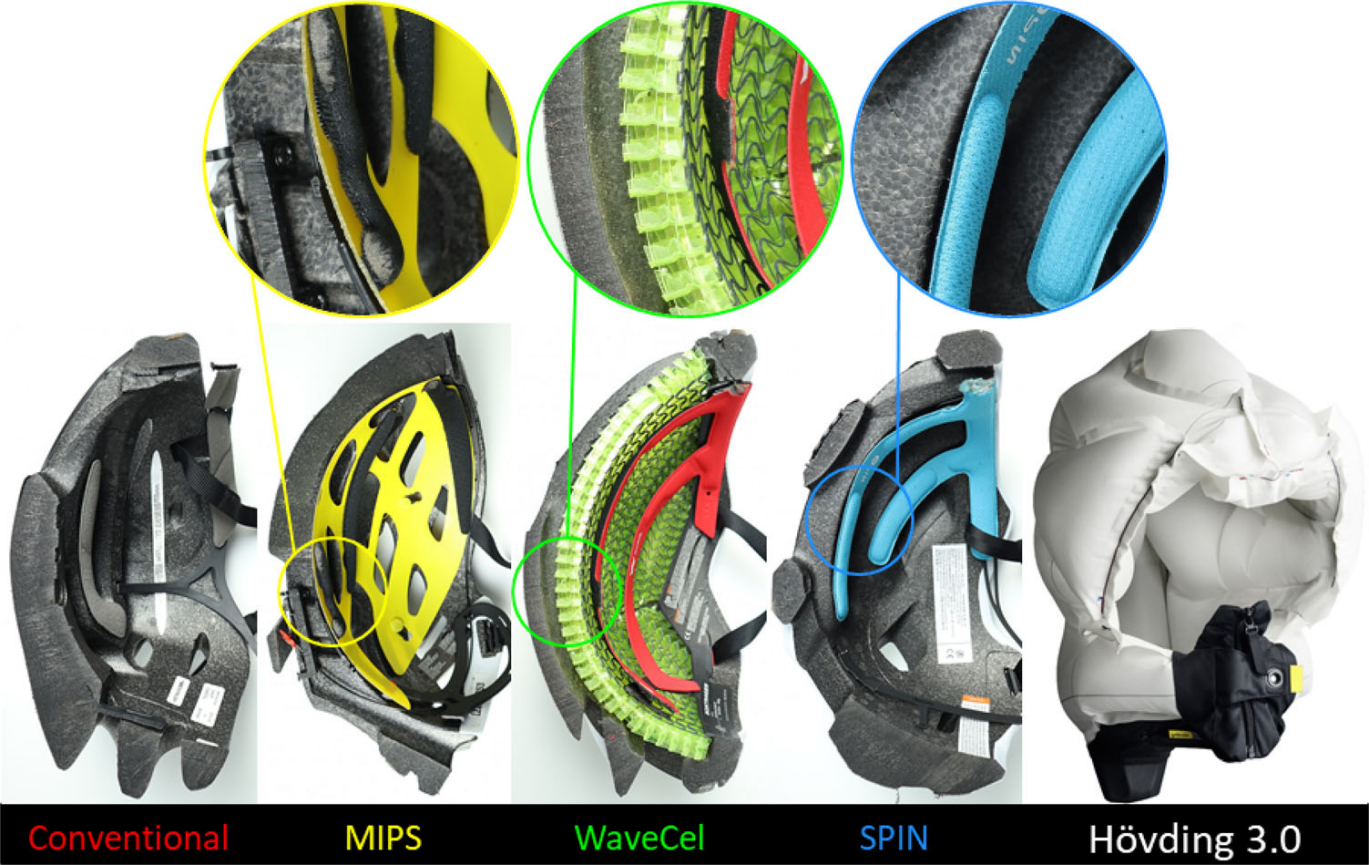
Mid-sagittal cross-sectional views of some of the helmets used in this study (from left to right): A conventional helmet, a ‘multi-directional impact protection system’ (MIPS) helmet, a corrugated ‘wavy’ cellular liner (WaveCel) helmet, a shear pad (SPIN) helmet and an airbag helmet (Hövding 3.0).

27 commercially available bicycle helmets were selected from the European market (both online and in-store), representing a large number of commonly used helmets. The price ranged from £10 to £275, reflecting a wide range of designs. Since we used a Hybrid III 50th percentile male dummy headform with a 58 cm circumference, we selected helmets with a size range that included 58 cm. Table 1 lists all the helmets and their rotational technology, if any. Helmets without a dedicated rotational technology are referred to as ‘conventional’ and serve as the controls for evaluating the effectiveness of the helmets incorporating rotational technologies. Four new technologies were investigated. 15 helmets were fitted with the ‘multi-directional impact protection system’ (MIPS)—a low-friction slip-layer that lies between the helmet liner and the head (Fig. 1) which enhances the decoupling between the helmet and head rotations.^23^ We also included a helmet with the corrugated ‘wavy’ cellular liner called WaveCel (Fig. 1). This liner technology is claimed to increase shear-compliance during collapse and mitigate head rotation.^8^ Another technology that we evaluated was the add-on shear pads, called SPIN (Fig. 1). This technology is also claimed to increase the relative motion between the helmet and head, thus mitigating head rotation. Finally, we included a radical technology, the airbag helmet Hövding 3.0 (Fig. 1).^32^ This helmet has been shown to reduce the head linear acceleration by several folds^32^ and has been shown to reduce peak head rotational acceleration in oblique impacts.^49^

**Table 1:** Summary of all the bicycle helmets included in the study with their respective technologies dedicated for managing rotational motion of the head in impact and advertised helmet type i.e. Urban/Skate, Road, or Mountain Bike (MTB)

Bike helmets	Helmet ID (HID)	Rotational technologies	Type
Abus Hyban 2	1	–	Urban/skate
Bell crest universal	7	–	Urban/skate
Biltema bicycle helmet	2	–	Road
Closa design fuga	24	–	Urban/skate
Giro caden	26	–	Urban/skate
Halford commuter helmet	12	–	Urban/skate
Rockrider MTB ST 500	20	–	MTB
Van Rysel RoadR 900	19	–	Road
Bell super air R	8	MIPS	MTB
Bell trace	10	MIPS	Road
Biltema bicycle helmet	15	MIPS	Road
Bontrager solstice	6	MIPS	Road
Giro agilis	17	MIPS	Road
Giro caden	29	MIPS	Urban/skate
Giro quarter FS	27	MIPS	Urban/skate
Lazer blade	3	MIPS	Road
Occano	28	MIPS	Road
Scott vivo plus	22	MIPS	MTB
Smith convoy	13	MIPS	Road
Specialized ambush ANGi MIPS	25	MIPS	MTB
Specialized S-works prevail II w/ ANGi MIPS	23	MIPS	Road
Sweet protection outrider	21	MIPS	Road
Tec quadriga	18	MIPS	Road
Bontrager specter WaveCel	4	WaveCel	Road
POC axion SPIN	14	SPIN	MTB
POC tectal SPIN	16	SPIN	MTB
Hövding 3.0	5	Airbag	Road

### Oblique Impact Tests

**Figure 2:**
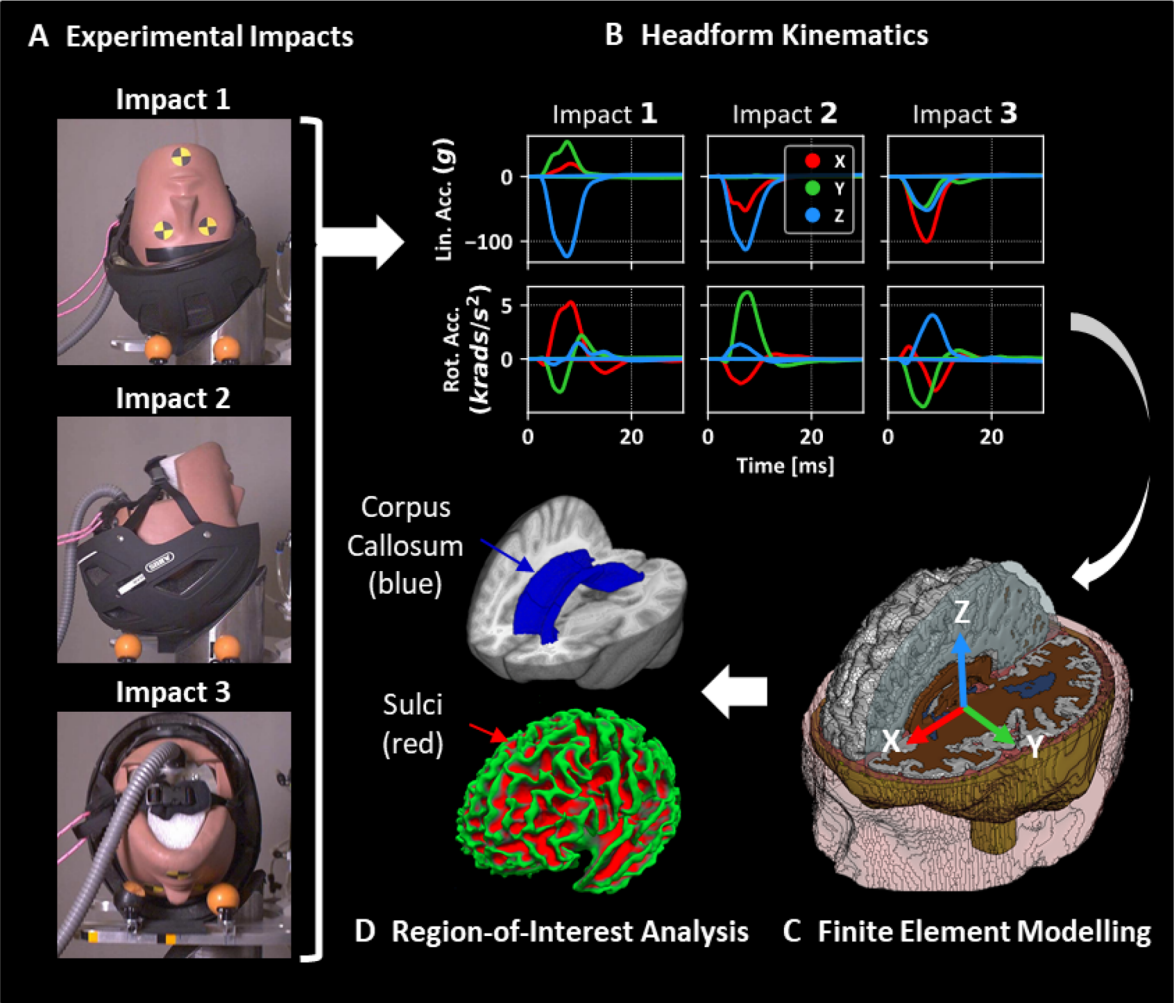
Setup of the three experimental impact conditions carried out for each helmet (**a**). For each of the impacts, three translational and three rotational acceleration time-history pulses are recorded about the CoG of the HIII headform (**b**). These are then applied to the detailed finite element model of TBI (**c**) which is then further analysed to extract brain strain as an injury metric in regions-of-interest such as the corpus callosum and sulci (**d**). The three impacts were selected to produce different head rotations (**a**, **b**). Impact 1, with the initial position of the headform *X*-, *Y*- and *Z*-axis 0°, produces predominant rotation about the *X*-axis. For impact 2, the initial position of the headform was *X*-, *Y*-axis 0° and *Z*-axis -90°, which produces predominant rotation about the *Y* axis. For impact 3, the Initial position of the headform was *X*- and *Z*-axis 0° and 65° around *Y*-axis. This impact produces large rotation about the *Z*-axis compared to the other impacts.

In order to test helmets under oblique impacts at different locations, we used the method proposed by the CEN Working Group 11 “Rotational test methods”.^54^ This method requires testing helmets under three different oblique impacts, shown in Fig. 2a. These impacts are representative of impacts in bicycle accidents and are based on the reconstruction of 1024 bicycle accidents.^10^,^54^ The helmet was mounted onto the 50th male Hybrid III headform, the chin strap was fastened and the helmeted headform was dropped onto a 45° anvil covered with a 40-grit sandpaper representing asphalt. The impact speed was 6.3 m/s. A digital inclinometer was used to position the helmeted headform, and a camera system was used to ensure the precision in positioning. An array of nine accelerometers in the 3-2-2-2 arrangement was mounted inside the headform.^43^ This method allowed us to determine the linear and rotational accelerations of the centre of gravity (CoG) of the headform with respect to the head-fixed axes (Fig. 2b and 2c). The accelerations were acquired at a frequency of 20kHz and filtered using an IOtechDBK4 12-pole Butterworth low-pass filter.^1^

Each helmet was tested at least twice for each impact location using two separate helmets to analyse the variability. The mean responses for all six accelerations were calculated and used for further analysis. All tests were performed by a test lab accredited for testing and certification in accordance with the European standard via Folksam Insurance Group. They were using the same test set-up as for the regulatory tests. Thereby, each helmet was inspected, and the impact locations were chosen to be far separated from prior impact location to minimize influence of prior damage in subsequent tests.

### Kinematics-Based Measures of TBI

The linear and rotational accelerations of the headform’s CoG were processed to extract the kinematic injury metrics that are commonly used to predict brain injury, including peak translational acceleration (PTA), peak rotational acceleration (PRA), peak rotational velocity (PRV) and brain injury criterion (BrIC) 50. The peak values are the maximum of the magnitude of each vector:

1 $${\text{PTA}} = \hbox{max} \left\{ {\sqrt {a_{x} \left( t \right)^{2} + a_{y} \left( t \right)^{2} + a_{z} \left( t \right)^{2} } } \right\}$$

2 $${\text{PRA}} = \hbox{max} \left\{ {\sqrt {\dot{\omega }_{x} \left( t \right)^{2} + \dot{\omega }_{y} \left( t \right)^{2} + \dot{\omega }_{z} \left( t \right)^{2} } } \right\}$$

3 $${\text{PRV}} = \hbox{max} \left\{ {\sqrt {\omega_{x} \left( t \right)^{2} + \omega_{y} \left( t \right)^{2} + \omega_{z} \left( t \right)^{2} } } \right\}$$

4 $${\text{BrIC}} = \sqrt { (\hbox{max} \{ \omega_{x} \left( t \right)\} /\omega_{xC} )^{2} + (\hbox{max} \{ \omega_{y} \left( t \right)\} /\omega_{yC} )^{2} + (\hbox{max} \{ \omega_{z} \left( t \right)\} /\omega_{zC} )^{2} } .$$

In these equations, $\vec{a} = \left( {a_{x} ,a_{y} ,a_{z} } \right)$ is the translational acceleration, $\vec{\omega }$ is the rotational velocity and $\dot{\vec{\omega }}$ is the rotational acceleration. For the calculation of BrIC, the peak angular velocities about each axis were employed regardless of the time at which each peak occurs, and $\omega_{xC}$, $\omega_{yC}$ and $\omega_{zC}$ are the components of the critical rotational velocity with values 66.25, 56.45 and 42.87 rads/s respectively as recommended by Takhounts *et al*.^50^

### Finite Element Modelling of TBI

We used an anatomically detailed finite element model of TBI to predict the distribution of strain across the brain during oblique impacts.^22^,^29^,^48^ The model incorporates fine details of brain anatomy such as sulci and gyri. The prediction of the model for brain displacement has been validated against recent cadaver experiments where the post-mortem human subject heads were subjected to well-controlled rotations.^21^

To simulate the impacts, the skull was assumed rigid due to its negligible deformation in helmeted impacts and the headform CoG accelerations from the experimental impacts were applied to the skull at the CoG of the head model (Figs. 2b and 2c). Simulations were carried out using the non-linear explicit dynamics solver LS-DYNA (R10.0, LSTC, US). Each simulation spanned 30 ms from the start of impact except for the Hövding impact which spanned 75 ms due to the extended time it remained in contact with the anvil. These durations ensured full capture of the peak brain deformation and strains resulting from the impact. The simulation outputs were postprocessed to determine the maximum value of the 1st principal Green-Lagrange strain at each element of the brain (called strain hereafter) and results were written into a NIFTI (Neuroimaging Informatics Technology Initiative) file for further analysis.

We determined the 90th percentile value of strain across the whole brain as a measure of overall brain response to the impact. We also determined strain in two regions of interest, corpus callosum and sulci (Fig. 2d). For the corpus callosum, the 90th percentile strain was determined. To determine strain in sulci, first Freesurfer was used to segment the structural MRI used to generate the FE model. This process resulted in an accurate spatial map of the grey/white matter boundary, which was then subdivided into regions of interest based on the Destrieux Atlas, including labelling of 30 gyri and 33 sulci in each hemisphere. The NIFTI image of strain was registered to the Freesurfer space using a standard affine transformation. This allowed for the calculation of mean strain within the anatomically correct sulcal maps.

### Statistical Analysis

The performance of each helmet fitted with a new technology was compared with the performance of conventional helmets serving as controls. The mean and standard deviation of the injury metrics of conventional helmets were used to calculate helmet-specific *z*-scores. A *z*-score of − 1 indicates that the performance measure of the helmet is one standard deviation smaller than the mean of the controls. We used a significance level of 0.05, which for a two-sided test is equivalent to a *z*-score outside the − 1.96 to + 1.96 range.^16^ Hence, a helmet that is significantly different to conventional helmets would have a *z*-score outside these bounds. We also determined the percentage change of each outcome measure of a helmet with respect to the mean of the conventional helmets.

## Results

### Head Motion

**Figure 3:**
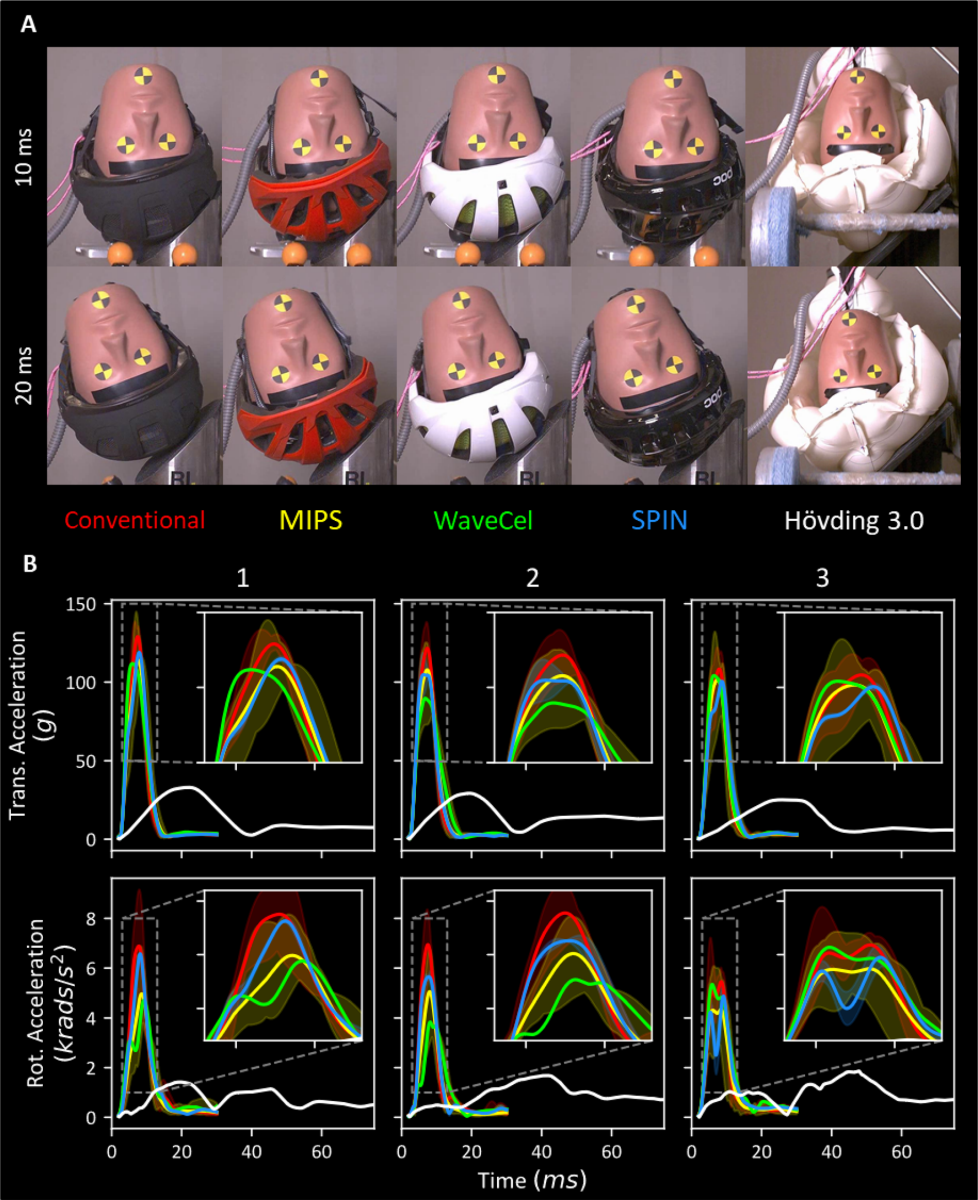
(**a**) Snapshots from the high-speed videos of a helmet from each technology category captured 10 ms apart, 10 and 20 ms after the start of impact 1. (**b**) Mean resultant translational and rotational time-history pulses colour-coded by technology. A filled region bounds the minimum and maximum recorded traces across all helmets where more than one helmet was assessed for a given technology. The results show that acceleration pulses peaked between 8 and 10 ms with an impact duration under 20 ms except for the Hövding 3.0 airbag helmet (white). Due to the larger size of the inflated airbag helmet, the impact duration was significantly increased and hence the peak was dramatically reduced. In all cases, translational accelerations did not exceed 150 g and rotational accelerations rarely exceeded 8 krads ms^−1^.

Snapshots of the high-speed videos for impact 1 are shown in Fig. 3a for some helmets along with the mean and bounds of the linear and rotational acceleration time histories for all impacts in Fig. 3b. Between 8 and 10 ms, when both linear and rotational accelerations have reached their peak, the helmets have rotated noticeably on the headform except the Hövding airbag helmet. At time 20 ms, headform accelerations have reached near zero for all helmets, except for Hövding, causing noticeable headform rotation (Fig. 3a).

The performance of the Hövding helmet is very different to the other helmets. It remains in contact with the anvil for 2–3 folds longer than the other helmets and as a result the acceleration of the headform is 2–3 folds smaller than that with other helmets across all impacts (Fig. 3b).

### Kinematics-Based Measures of Brain Injury Are Lower in Helmets with New Technologies

**Figure 4:**
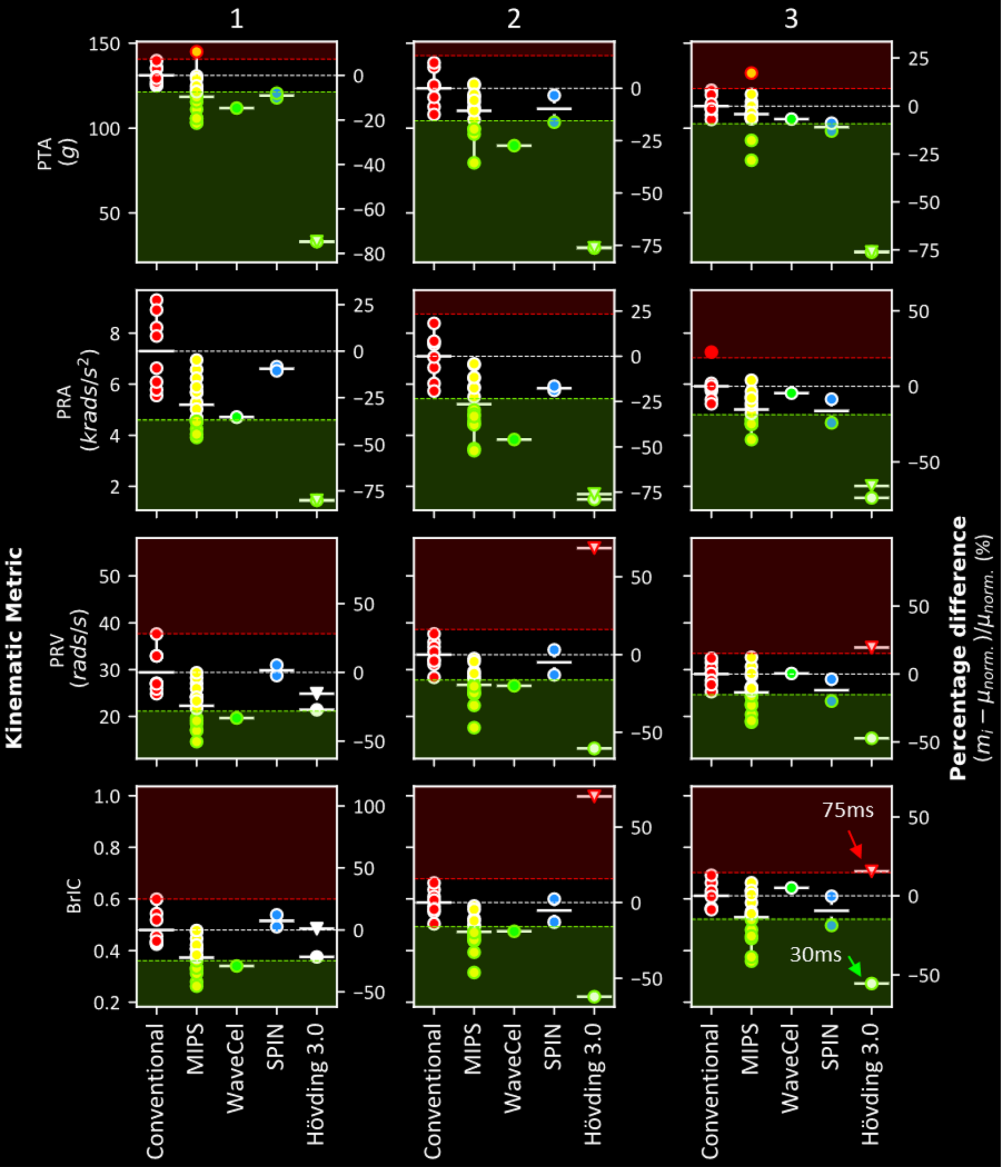
The performance of all the helmets in impact condition 1 (left), 2 (middle) and 3 (right) grouped by technology (marker fill colour) with respect to the four kinematic metrics assessed (PTA, PRA, PRV and BrIC). Solid white horizontal lines represent the mean metric value of each technology. The dotted white horizontal lines represent the mean for conventional helmets (red). The red and green margins represent regions where the performance would be significantly worse or better than conventional helmets for that metric (*p* < 0.05). The right-hand side axis of each plot represents the percentage difference of the metric value of each helmet with respect to the mean of the conventional helmets. The results show that, in most cases, helmets perform either significantly better than (green marker edge) or insignificantly different to conventional helmets, with rare occasions where helmets perform significantly worse (red marker edge).

Some of the helmets fitted with the new technologies had significantly different PTA, PRA, PRV and BrIC compared to conventional helmets (Fig. 4). The helmets fitted with MIPS had significantly lower PTA compared to conventional helmets for all impacts (impact 1: 7–21%, 2: 19–36%, 3: 18–28% - for *z*-scores and *p* values please see Tables 2, 3, 4, 5). However, one of the helmets with MIPS had a significantly higher PTA for impact 1 (11%) and another MIPS helmet had a significantly higher PTA for impact 3 (17%). 33% of the helmets fitted with MIPS had significantly lower PRA in impact 1 (38–46%), 53% in impact 2 (30–52%) and 40% in impact 3 (22–35%). Similarly, 40% of the helmets fitted with MIPS had significantly lower PRV in impact 1 (33–50%), 60% in impact 2 (16–47%) and 47% in impact 3 (16–35%). Finally, 47% of the helmets fitted with MIPS had significantly lower BrIC in impact 1 (25–45%), 60% in impact 2 (16–46%) and 40% in impact 3 (15–41%). When comparing the Giro Caden helmet versions with and without MIPS, we find that all kinematic-based injury metrics are reduced with the MIPS version (Tables 1, 2, 3, 4, 5). When comparing the Biltema helmet versions with and without MIPS, we find that all kinematic-based injury metrics are reduced with the MIPS version except for PTA, which was increased. However, the Biltema helmet versions had design differences not exclusive to MIPS. None of the helmets fitted with MIPS had significantly higher rotational measures of brain injury compared to the conventional helmets.

**Table 2:** The PTA percentage difference, *z*-score and *p* value of each helmet (referenced by the HID) in comparison to the conventional helmets for each impact condition. The HID cell colours represent the different technologies (see Fig. 1).

*PTA*	Impact 1			Impact 2			Impact 3		
HID	% diff.	*z*-score	*p* value	% diff.	*z*-score	*p* value	% diff.	*z*-score	*p* value
3	− 9	− 2.416	0.016	1.4	0.18	0.857	2.3	0.482	0.63
6	− 7.4	− 1.985	0.047	− 9.8	− 1.242	0.214	0.2	0.035	0.972
8	− 20.9	− 5.591	0	− 3.8	− 0.475	0.635	− 0.1	− 0.012	0.99
10	− 6.1	− 1.632	0.103	1.9	0.239	0.811	− 4.5	− 0.948	0.343
13	10.7	2.85	0.004	− 6.8	− 0.862	0.389	− 5.8	− 1.228	0.219
15	− 0.3	− 0.077	0.939	− 4.3	− 0.539	0.59	17.1	3.641	0
17	− 15.2	− 4.061	0	− 2.9	− 0.368	0.713	− 4.8	− 1.02	0.308
18	− 19.3	− 5.16	0	− 19.4	− 2.447	0.014	6.1	1.307	0.191
21	− 21.4	− 5.72	0	− 21.9	− 2.767	0.006	− 17.9	− 3.807	0
22	− 11.8	− 3.15	0.002	− 14.9	− 1.888	0.059	− 6	− 1.273	0.203
23	− 19.2	− 5.14	0	− 35.6	− 4.501	0	− 28.3	− 6.017	0
25	− 9.6	− 2.571	0.01	− 9.1	− 1.145	0.252	− 6.7	− 1.415	0.157
27	− 1.5	− 0.408	0.683	− 10.7	− 1.348	0.178	− 4.7	− 1.003	0.316
28	− 4.7	− 1.266	0.206	− 19.5	− 2.463	0.014	− 3.6	− 0.763	0.445
29	− 7.2	− 1.93	0.054	− 5.7	− 0.727	0.467	− 6.3	− 1.351	0.177
4	− 14.6	− 3.905	0	− 27.4	− 3.463	0.001	− 6.8	− 1.441	0.15
14	− 10	− 2.669	0.008	− 3.4	− 0.428	0.669	− 13	− 2.774	0.006
16	− 7.9	− 2.125	0.034	− 16.2	− 2.047	0.041	− 8.9	− 1.899	0.058
5_30 ms_	− 74.8	− 19.984	0	− 76.2	− 9.632	0	− 76.1	− 16.205	0
5_75 ms_	− 74.8	− 19.984	0	− 76.2	− 9.632	0	− 76.1	− 16.205	0

**Table 3:** The PRA percentage difference, *z*-score and *p* value of each helmet (referenced by the HID) in comparison to the conventional helmets for each impact condition. The HID cell colours represent the different technologies (see Fig. 1).

*PRA*	Impact 1			Impact 2			Impact 3		
HID	% diff.	*z*-score	*p* value	% diff.	*z*-score	*p* value	% diff.	*z*-score	*p* value
3	− 27.6	− 1.466	0.143	− 17	− 1.423	0.155	− 35.2	− 3.658	0
6	− 35.9	− 1.906	0.057	− 22.4	− 1.877	0.061	− 14.9	− 1.545	0.122
8	− 46.3	− 2.458	0.014	− 37	− 3.101	0.002	− 25.2	− 2.617	0.009
10	− 41.8	− 2.216	0.027	− 35.4	− 2.966	0.003	− 21.7	− 2.257	0.024
13	− 9.9	− 0.526	0.599	− 4.7	− 0.395	0.693	− 6.5	− 0.676	0.499
15	− 31.2	− 1.655	0.098	− 50.9	− 4.265	0	− 22.3	− 2.312	0.021
17	− 14.6	− 0.773	0.44	− 12.1	− 1.016	0.31	− 7.9	− 0.824	0.41
18	− 37.8	− 2.007	0.045	− 29.7	− 2.49	0.013	− 24.2	− 2.517	0.012
21	− 4.8	− 0.253	0.8	− 37.7	− 3.156	0.002	− 24.8	− 2.574	0.01
22	− 36.5	− 1.936	0.053	− 32	− 2.682	0.007	− 17.9	− 1.856	0.063
23	− 37.8	− 2.006	0.045	− 52.1	− 4.361	0	− 12.6	− 1.309	0.191
25	− 22.4	− 1.19	0.234	− 17.6	− 1.47	0.142	− 11.1	− 1.149	0.251
27	− 21.9	− 1.163	0.245	− 11.6	− 0.974	0.33	4	0.412	0.68
28	− 44.5	− 2.363	0.018	− 33.2	− 2.777	0.005	− 3.5	− 0.365	0.715
29	− 19.2	− 1.02	0.308	− 4.2	− 0.35	0.726	− 7.9	− 0.82	0.412
4	− 35.4	− 1.88	0.06	− 45.9	− 3.845	0	− 4.6	− 0.475	0.635
14	− 8.4	− 0.443	0.658	− 18.8	− 1.576	0.115	− 24.1	− 2.506	0.012
16	− 10.6	− 0.563	0.573	− 16.6	− 1.387	0.165	− 8.6	− 0.89	0.373
5_30 ms_	− 74.8	− 19.984	0	− 76.2	− 9.632	0	− 76.1	− 16.205	0
5_75 ms_	− 74.8	− 19.984	0	− 76.2	− 9.632	0	− 76.1	− 16.205	0

**Table 4:** The PRV percentage difference, *z-* score and *p* value of each helmet (referenced by the HID) in comparison to the conventional helmets for each impact condition. The HID cell colours represent the different technologies (see Fig 1).

*PRV*	Impact 1			Impact 2			Impact 3		
HID	% diff.	*z-* score	*p* value	% diff.	*z-* score	*p* value	% diff.	*z-* score	*p* value
3	− 17.6	− 1.226	0.22	− 22.5	− 2.705	0.007	− 29.8	− 3.845	0
6	− 33.1	− 2.304	0.021	− 16.3	− 1.964	0.05	− 22	− 2.836	0.005
8	− 17.8	− 1.238	0.216	− 24.5	− 2.954	0.003	− 17	− 2.194	0.028
10	− 41.6	− 2.897	0.004	− 32.7	− 3.938	0	− 23	− 2.968	0.003
13	− 8	− 0.56	0.575	− 2.5	− 0.304	0.761	− 9.5	− 1.222	0.222
15	− 37.3	− 2.597	0.009	− 47	− 5.662	0	− 35.5	− 4.58	0
17	− 4	− 0.276	0.783	− 13.8	− 1.667	0.096	− 10	− 1.29	0.197
18	− 42.4	− 2.955	0.003	− 15.3	− 1.842	0.065	− 34.3	− 4.429	0
21	− 0.2	− 0.017	0.986	− 25.5	− 3.065	0.002	− 16	− 2.067	0.039
22	− 35.2	− 2.451	0.014	− 24.5	− 2.95	0.003	− 12.3	− 1.593	0.111
23	− 26.2	− 1.823	0.068	− 19.7	− 2.376	0.018	5.4	0.695	0.487
25	− 11.7	− 0.814	0.416	− 10.8	− 1.303	0.193	− 10.5	− 1.358	0.174
27	− 18.1	− 1.26	0.208	− 4.4	− 0.532	0.595	12.3	1.583	0.113
28	− 50.4	− 3.508	0	− 20.3	− 2.449	0.014	− 1.2	− 0.16	0.873
29	− 20.8	− 1.45	0.147	− 12.2	− 1.464	0.143	0.4	0.051	0.959
4	− 33.2	− 2.312	0.021	− 20	− 2.414	0.016	0.4	0.052	0.959
14	− 2.3	− 0.161	0.872	− 13	− 1.57	0.116	− 20	− 2.578	0.01
16	5.3	0.37	0.711	3.1	0.376	0.707	− 3.8	− 0.496	0.62
5_30 ms_	− 27.2	− 1.894	0.058	− 60.4	− 7.271	0	− 47.3	− 6.102	0
5_75 ms_	− 15.5	− 1.077	0.281	68.5	8.248	0	19.5	2.517	0.012

**Table 5:** The BrIC percentage difference, z- score and p− value of each helmet (referenced by the HID) in comparison to the conventional helmets for each impact condition. The HID cell colours represent the different technologies (see Fig. 1).

*BrIC*	Impact 1			Impact 2			Impact 3		
HID	% diff.	*z-* score	*p* value	% diff.	*z-* score	*p* value	% diff.	*z-* score	*p* value
3	− 15.5	− 1.224	0.221	− 22.8	− 2.837	0.005	− 26.9	− 3.575	0
6	− 30.8	− 2.431	0.015	− 16.1	− 2.005	0.045	− 21.4	− 2.847	0.004
8	− 15.2	− 1.204	0.229	− 21.6	− 2.691	0.007	− 12.8	− 1.707	0.088
10	− 41.8	− 3.304	0.001	− 32.8	− 4.076	0	− 25.4	− 3.37	0.001
13	− 1.4	− 0.11	0.912	− 2.3	− 0.287	0.774	− 4.3	− 0.578	0.563
15	− 32.5	− 2.57	0.01	− 46.2	− 5.744	0	− 41.3	− 5.486	0
17	− 7	− 0.554	0.58	− 14.6	− 1.812	0.07	− 12.4	− 1.644	0.1
18	− 35.2	− 2.776	0.006	− 15.6	− 1.935	0.053	− 38.6	− 5.136	0
21	− 0.4	− 0.031	0.975	− 25.2	− 3.131	0.002	− 14.9	− 1.974	0.048
22	− 30.6	− 2.42	0.016	− 24.4	− 3.039	0.002	− 10.1	− 1.34	0.18
23	− 24.9	− 1.965	0.049	− 19.5	− 2.422	0.015	2.9	0.392	0.695
25	− 12.2	− 0.961	0.337	− 10.7	− 1.328	0.184	− 11.3	− 1.507	0.132
27	− 21.9	− 1.73	0.084	− 4.6	− 0.572	0.567	8.2	1.094	0.274
28	− 45.4	− 3.586	0	− 19.7	− 2.452	0.014	3.3	0.437	0.662
29	− 20.2	− 1.593	0.111	− 12	− 1.49	0.136	0.7	0.096	0.924
4	− 29.1	− 2.298	0.022	− 18.9	− 2.347	0.019	5	0.669	0.503
14	2.6	0.208	0.835	− 12.8	− 1.595	0.111	− 18.6	− 2.476	0.013
16	− 7.9	− 2.125	0.034	− 16.2	− 2.047	0.041	− 8.9	− 1.899	0.058
5_30 ms_	− 74.8	− 19.984	0	− 76.2	− 9.632	0	− 76.1	− 16.205	0
5_75 ms_	− 74.8	− 19.984	0	− 76.2	− 9.632	0	− 76.1	− 16.205	0

In comparison to conventional helmets, the WaveCel helmet had significantly lower PTA in impacts 1 and 2 (1: 15% and 2: 27%). This helmet also had significantly reduced PRV in impact 1, and PRA and PRV in impact 2 (1: 33% reduction in PRV, 2: 46% reduction in PRA and 20% reduction in PRV). The WaveCel helmet had a significantly lower BrIC in impact 1 and 2 (1: 29%, 2: 19%).

The helmets fitted with SPIN (Axion and Tectal), hereon referred to as SPIN 1 and SPIN 2 respectively, presented different responses. Only SPIN 1 had a significantly lower PTA in impact 1 and 3 compared to conventional helmets (1: 10%, 3: 13%). However, only SPIN 2 had a significantly lower PTA in impact 2 (16%). Neither SPIN helmets had significantly lower PRA, PRV or BrIC in impact 1 and 2. Only SPIN 1 had a significantly lower PRA, PRV and BrIC in impact 3 (PRA: 24%, PRV: 20%, BrIC: 19%).

Finally, the airbag helmet had significantly reduced PTA and PRA in all impacts considering both 30 and 75 ms durations. PTA was significantly reduced in all three impacts with this helmet irrespective of duration (1: 75%, 2: 76% and 3: 76%). PRA was also significantly reduced almost identically across all three impacts. However, the reduction in PRA slightly depended on analysis duration particularly for impact 3 (1: 80%, 2: 76% and 3: 66% @ 30ms; 1: 80%, 2: 79% and 3: 74% @ 75ms). PRV and BrIC were more dramatically affected by analysis duration. We found both PRV and BrIC to be significantly larger with this helmet in impact 2 (PRV: 69%, BrIC: 70%) and 3 (PRV: 20%, BrIC: 16%) and within normal ranges in impact 1 considering a 75ms analysis duration. Considering a 30ms duration, PRV and BrIC were significantly reduced in both impact 2 (PRV: 60%, BrIC: 62%) and 3 (PRV: 47%, BrIC: 55%) and within nominal ranges in impact 1.

### Strain Across the Whole Brain Is Lower in Helmets with New Technologies

**Figure 5:**
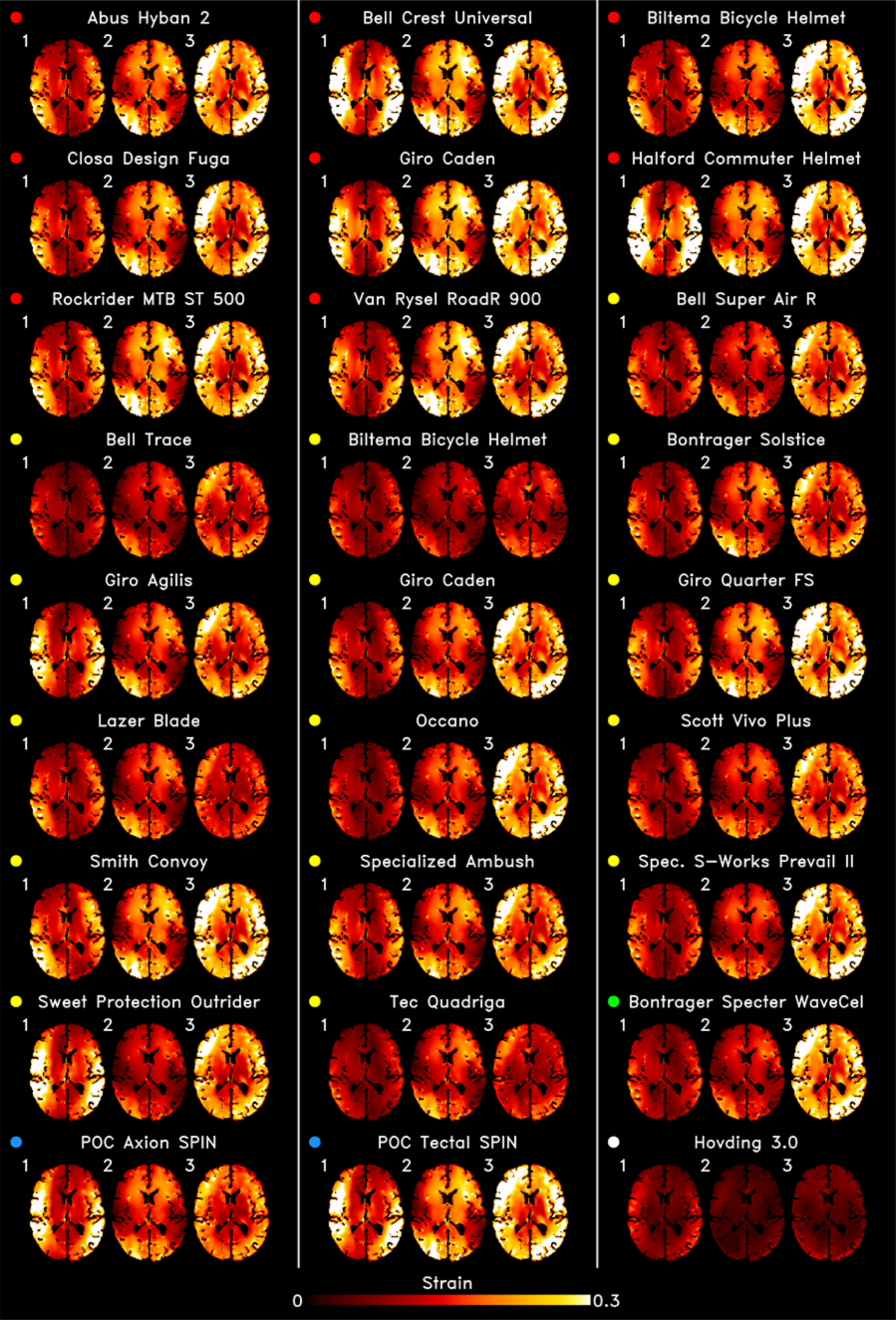
Voxel-wise representation of the maximum Green Lagrange strain in the brain in the transverse plane as a result of each impact condition for each helmet. The coloured marker to the left of each helmet name represents the technology. The results show a large variation in brain strain patterns across helmets and across impact conditions for each helmet.

We observed a large variation in strain distribution across the brain when using different helmets in impacts 1, 2 and 3 (Fig. 5). The axial sections of the brain show that the three impact conditions led to noticeably different strain patterns for each helmet. Generally, larger strains where more focused in the cortical regions and the corpus callosum.

**Figure 6:**
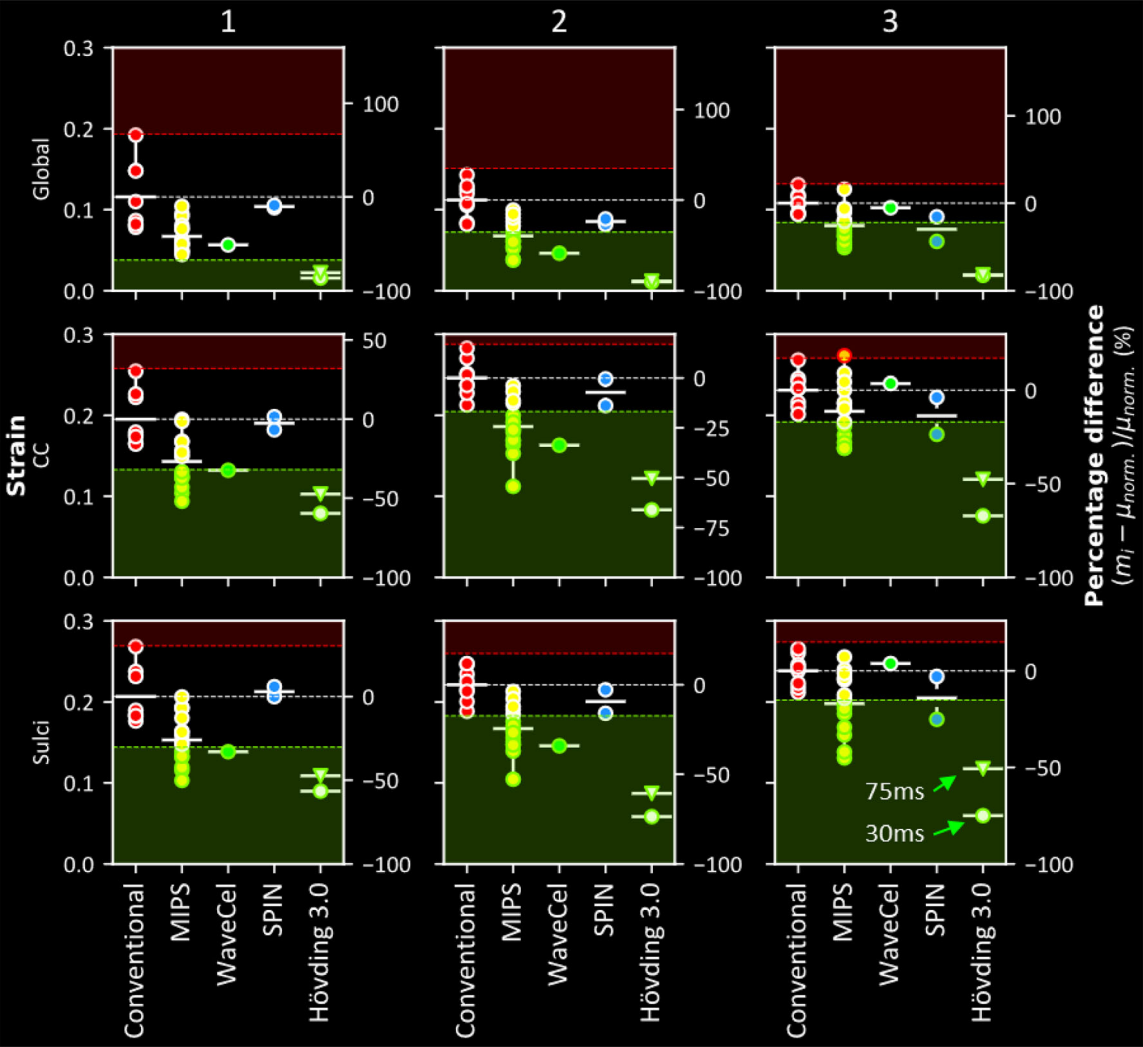
The performance of all the helmets in impact condition 1 (left), 2 (middle) and 3 (right) grouped by technology (marker fill colour) with respect to the Green-Lagrange strain across the entire brain (global) as well as in brain regions-of-interest (corpus callosum and sulci). For the global and corpus callosum, the 90th percentile strain value was used. For the sulci, the mean strain value was used. Solid white horizontal lines represent the mean metric value of each technology. The dotted white horizontal lines represent the mean for conventional helmets (red). The red and green margins represent regions where the performance would be significantly worse or better than conventional helmets for that metric (*p* > 0.05). The right-hand side axis of each plot represents the percentage difference of the metric value of each helmet with respect to the mean of the conventional helmets. The results show that, in most cases, helmets perform either significantly better than (green marker edge) or insignificantly different to conventional helmets, with rare occasions where helmets perform significantly worse (red marker edge).

To better show the effects of the technology on brain strain, we plotted the 90th percentile strain across the whole brain in Fig. 6. A large variation in the strain can be seen across the helmets, from 0.01 with the airbag helmet to 0.19 with a conventional helmet. Compared to the performance of conventional helmets, strain was within normal ranges in all MIPS helmets for impact 1. In impacts 2 and 3, strain was significantly lower in 60% of MIPS helmets (2: 35–66%, 3: 23–51% - for *z*-scores and *p* values please see Tables 6). When comparing the Giro Caden and Biltema helmet versions with and without MIPS, we find that strain measures in all brain regions are reduced with the MIPS versions.

**Table 6:** The global 90th percentile brain strain percentage difference, *z-* score and *p* value of each helmet (referenced by the HID) in comparison to the conventional helmets for each impact condition. The HID cell colours represent the different technologies (see Fig. 1).

*Global*	Impact 1			Impact 2			Impact 3		
HID	% diff.	*z-* score	*p* value	% diff.	*z-* score	*p* value	% diff.	*z-* score	*p* value
3	− 45	− 1.313	0.189	− 34.7	− 1.938	0.053	− 50.7	− 4.491	0
6	− 49.7	− 1.45	0.147	− 35.3	− 1.973	0.048	− 31.2	− 2.768	0.006
8	− 58.7	− 1.713	0.087	− 51.8	− 2.892	0.004	− 37.8	− 3.348	0.001
10	− 57.3	− 1.673	0.094	− 50.5	− 2.82	0.005	− 34.1	− 3.022	0.003
13	− 18.3	− 0.534	0.593	− 11.1	− 0.622	0.534	− 10.1	− 0.898	0.369
15	− 49.2	− 1.436	0.151	− 65.1	− 3.633	0	− 47	− 4.164	0
17	− 20.9	− 0.609	0.543	− 24.1	− 1.347	0.178	− 22.8	− 2.018	0.044
18	− 57.4	− 1.676	0.094	− 43.6	− 2.437	0.015	− 44.3	− 3.926	0
21	− 10	− 0.292	0.77	− 53.2	− 2.97	0.003	− 37.2	− 3.294	0.001
22	− 52.1	− 1.52	0.129	− 45.3	− 2.531	0.011	− 28.8	− 2.547	0.011
23	− 50	− 1.458	0.145	− 66.2	− 3.7	0	− 19.4	− 1.717	0.086
25	− 30.3	− 0.884	0.377	− 28.5	− 1.589	0.112	− 20.7	− 1.829	0.067
27	− 33.7	− 0.983	0.326	− 19.8	− 1.107	0.268	16	1.417	0.156
28	− 61.3	− 1.788	0.074	− 45.9	− 2.565	0.01	− 8.5	− 0.754	0.451
29	− 33.9	− 0.989	0.323	− 15.6	− 0.873	0.383	− 6.3	− 0.557	0.578
4	− 51	− 1.487	0.137	− 58.5	− 3.267	0.001	− 5.3	− 0.469	0.639
14	− 11.6	− 0.339	0.735	− 26.7	− 1.49	0.136	− 43.5	− 3.853	0
16	− 9.1	− 0.265	0.791	− 20.9	− 1.166	0.244	− 15.6	− 1.381	0.167
5_30 ms_	− 86.4	− 2.522	0.012	− 90.2	− 5.035	0	− 82.4	− 7.301	0
5_75 ms_	− 80.7	− 2.355	0.019	− 89.4	− 4.992	0	− 81.8	− 7.248	0

Likewise, strain was lower in the WaveCel helmet in impact 2 (59%), but not in impact 1 or 3. Strain was also significantly reduced in impact 3 in the SPIN 1 helmet, however no significant reductions were observed for the other impacts nor in the SPIN 2 helmet. The airbag helmet significantly reduced the strain in all three impacts irrespective of analysis duration (1: 86, 2: 90% and 3: 82% @ 30 ms and 1: 81, 2: 89% and 3: 82% @ 75ms). None of the helmets fitted with the new technologies showed increased global strain compared to the conventional helmets.

### Strain in Corpus Callosum Is Lower in Helmets with New Technologies

The 90th percentile strain in corpus callosum (CC) was generally larger than that across the whole brain, ranging from 0.08 to 0.28. Most helmets with new technologies had significantly lower CC strains. On one occasion, a helmet showed a significantly higher CC strain. Significantly lower strain in the CC was found with 47% of MIPS fitted helmets in impact 1 (33–52%), 73% in impact 2 (19–54%) and 40% in impact 3 (17–31% - for z-scores and p-values please see Tables 7). A significantly increased CC strain was found with one of the helmets fitted with MIPS in impact 3 (18%). Significantly lower CC strain was found with the WaveCel helmet in impact 1 and 2 (1: 32%, 2: 34%), but not 3. Of the SPIN helmets, a significant reduction in CC strain was found only with SPIN 1 in impact 3 (24%). A significant reduction in the CC strain was found in all impacts with the Hövding 3.0 helmet considering both analysis durations (1: 60%, 2: 66% and 3: 67% @ 30ms and 1: 48%, 2: 51% and 3: 48% @ 75ms,).

**Table 7:** The CC 90th percentile brain strain percentage difference, *z-* score and *p* value of each helmet (referenced by the HID) in comparison to the conventional helmets for each impact condition. The HID cell colours represent the different technologies (see Fig. 1).

*CC*	Impact 1			Impact 2			Impact 3		
HID	% diff.	*z-* score	*p* value	% diff.	*z-* score	*p* value	% diff.	*z-* score	*p* value
3	− 19.9	− 1.218	0.223	− 26.2	− 3.047	0.002	− 31.1	− 3.59	0
6	− 35.8	− 2.195	0.028	− 22.1	− 2.575	0.01	− 24.1	− 2.779	0.005
8	− 22.6	− 1.387	0.165	− 33.5	− 3.899	0	− 17.7	− 2.04	0.041
10	− 46.8	− 2.871	0.004	− 37.9	− 4.413	0	− 19.2	− 2.212	0.027
13	− 0.1	− 0.008	0.994	− 3.9	− 0.453	0.651	− 7.2	− 0.826	0.409
15	− 36.9	− 2.263	0.024	− 54.4	− 6.339	0	− 28	− 3.227	0.001
17	− 13.9	− 0.853	0.394	− 18.7	− 2.182	0.029	− 6.6	− 0.761	0.447
18	− 43	− 2.639	0.008	− 22.2	− 2.581	0.01	− 31	− 3.571	0
21	− 1.4	− 0.087	0.931	− 31.8	− 3.704	0	− 16.9	− 1.953	0.051
22	− 37.3	− 2.286	0.022	− 27.2	− 3.171	0.002	− 10	− 1.151	0.25
23	− 33.3	− 2.039	0.041	− 31.1	− 3.616	0	8.9	1.03	0.303
25	− 14.3	− 0.878	0.38	− 12.6	− 1.472	0.141	− 9.8	− 1.129	0.259
27	− 23.7	− 1.452	0.147	− 6.8	− 0.795	0.427	18.3	2.107	0.035
28	− 52	− 3.188	0.001	− 26	− 3.032	0.002	0.1	0.006	0.995
29	− 21.1	− 1.294	0.196	− 11.3	− 1.317	0.188	4.4	0.506	0.613
4	− 32.4	− 1.986	0.047	− 33.8	− 3.933	0	3.5	0.405	0.685
14	− 6.7	− 0.413	0.68	− 13.9	− 1.614	0.107	− 23.6	− 2.725	0.006
16	1.6	0.097	0.923	− 0.6	− 0.065	0.948	− 3.9	− 0.447	0.655
5_30 ms_	− 59.7	− 3.659	0	− 66.2	− 7.712	0	− 67.2	− 7.751	0
5_75 ms_	− 47.5	− 2.91	0.004	− 50.5	− 5.878	0	− 47.7	− 5.502	0

### Strain in Sulci Is Also Lower in Helmets with New Technologies

Finally, we determined the mean strain across all sulcal regions for all helmeted headform impacts. Strain in sulci was ranging from 0.06 to 0.27 and it was generally larger than the 90th percentile strain across the whole brain. Significant reduction in sulci strain was found with 40% of MIPS fitted helmets in impact 1 (33–50%), 67% in impact 2 (20–53%) and 53% in impact 3 (16–45% - for *z*-scores and *p* values please see Table 8). Significantly lower sulci strain was found with the WaveCel helmet in impacts 1 and 2 (1: 33%, 2: 34%) but not 3. Of the SPIN helmets, the sulci strain was significantly lower only with SPIN 1 in impact 3 (25%). A significant reduction in the sulci strain was found with the airbag helmet in all impacts irrespective of analysis duration (1: 57%, 2: 74% and 3: 75% @ 30 ms; 1: 47%, 2: 61% and 3: 51% @ 75 ms). None of the helmets fitted with new technologies showed significantly increased sulcal strain compared to the conventional helmets.

**Table 8:** The mean brain sulci strain percentage difference, *z-* score and *p* value of each helmet (referenced by the HID) in comparison to the conventional helmets for each impact condition. The HID cell colours represent the different technologies (see Fig. 1).

*Sulci*	Impact 1			Impact 2			Impact 3		
HID	% diff.	*z-* score	*p* value	% diff.	*z-* score	*p* value	% diff.	*z-* score	*p* value
3	− 23.4	− 1.515	0.13	− 25.8	− 2.915	0.004	− 33.1	− 4.331	0
6	− 32.7	− 2.12	0.034	− 20.4	− 2.312	0.021	− 22.1	− 2.886	0.004
8	− 27.6	− 1.788	0.074	− 33.2	− 3.758	0	− 17.8	− 2.322	0.02
10	− 44.2	− 2.865	0.004	− 37.1	− 4.191	0	− 29.1	− 3.807	0
13	− 6.5	− 0.424	0.672	− 3.7	− 0.424	0.672	− 4.5	− 0.593	0.553
15	− 36.3	− 2.354	0.019	− 52.8	− 5.971	0	− 45.1	− 5.892	0
17	− 7	− 0.452	0.651	− 16.3	− 1.84	0.066	− 16.5	− 2.151	0.031
18	− 42.3	− 2.741	0.006	− 22.8	− 2.573	0.01	− 42	− 5.481	0
21	− 0.2	− 0.015	0.988	− 31.7	− 3.585	0	− 19.5	− 2.541	0.011
22	− 35.6	− 2.311	0.021	− 29.5	− 3.338	0.001	− 14.1	− 1.848	0.065
23	− 28.6	− 1.852	0.064	− 33.7	− 3.808	0	− 3.4	− 0.45	0.653
25	− 12.8	− 0.832	0.405	− 13.9	− 1.572	0.116	− 12.3	− 1.612	0.107
27	− 20.9	− 1.353	0.176	− 7.7	− 0.869	0.385	7.2	0.941	0.347
28	− 50.2	− 3.253	0.001	− 26.7	− 3.015	0.003	0.7	0.085	0.932
29	− 21.3	− 1.382	0.167	− 12.2	− 1.378	0.168	− 1.2	− 0.161	0.872
4	− 33	− 2.141	0.032	− 34.1	− 3.86	0	3.8	0.496	0.62
14	− 0.1	− 0.007	0.994	− 15.9	− 1.803	0.071	− 25	− 3.27	0.001
16	5.9	0.382	0.702	− 2.8	− 0.314	0.754	− 2.9	− 0.373	0.709
5_30 ms_	− 56.6	− 3.671	0	− 73.7	− 8.334	0	− 75.1	− 9.806	0
5_75 ms_	− 47.4	− 3.074	0.002	− 60.7	− 6.865	0	− 50.7	− 6.622	0

## Discussion

We showed that the new helmet technologies can provide better protection under oblique impacts than conventional helmets. For this assessment, we used a new test method proposed by CEN/TC158/WG11, designed to represent real-world oblique impacts and recorded translational and rotational motions of the headform. This enabled a unique brain strain analysis which considers key anatomical regions such as corpus collosum and sulci using a highly detailed TBI model. The results of this study show that in comparison with the conventional helmets, the helmets fitted with MIPS, WaveCel, SPIN and Hövding can reduce peak rotational acceleration and velocity, BrIC, overall brain strain and strain in corpus callosum and sulci. None of these helmets showed a significant increase across all measures of injury compared to the conventional helmets, except for two helmets fitted with MIPS and the Hövding when considering a 75 ms analysis duration.

Our results show that the effectiveness of helmets in comparison with the conventional helmets depends on their technology, impact location and injury metric. For example, the number of MIPS helmets that were more effective than conventional helmets depends on the impact location. The discrepancy in performance of the helmets across impact locations could be due to the various thicknesses of the helmets in the different impact locations which may affect the resultant force vector and subsequent head motion.^36^ Another potential reason for the discrepancy between impact conditions is the geometric shape of the headform which is not symmetric about all anatomical planes. A helmet may rotate easier with less constraints imposed by the head in the coronal plane (impact 1) than the transverse plane (impact 3). A similar case may be found when comparing the coronal to the mid-sagittal rotations (impact 1 vs. impact 2). This may explain why helmets with MIPS and WaveCel were less effective in impacts 2 and 3 than 1 with respect to rotational kinematic and brain injury metrics. For the WaveCel helmet, its anisotropic liner design may also contribute to the different performances. Although cadaveric studies assessing the effect of the direction of rotational acceleration on TBI is limited, a few computational studies have shown that the brain tissue strain and TBI likelihood resulting from rotational acceleration in the transverse plane (axial rotation) can be larger than strain resulting from rotation in other anatomical planes.^5^,^50^,^53^ Hence, considering the poorer performance of some of the helmets with dedicated rotational damping systems in impact 3 than impacts 1 and 2, the performance of the helmets in future should better address rotations in the transverse plane.

These findings support the use of three different impact locations such as in this study, in contrast to previous work that has considered one impact location to compare injury mitigation of helmets.^9^ The choice of these locations is also an important one. We employed three oblique impact locations based on the method proposed by the CEN/TC158/WG11, which was derived from a head impact location probability map of 1024 cyclist falls.^10^,^54^ Our results provide further evidence as to why future standard methods designed to assess the mitigation effects of helmets on rotational motion of the head should include several impact locations.

A recent study introducing a novel comparable method for assessing helmets, named Summation of Tests for the Assessment of Risk (STAR), reinforces the finding of this study.^6^ The STAR method summarises the performance of a helmet into a single value based on head kinematics and concussion risk curves derived from American football players. Although the study used a National Operating Committee on Standards for Athletic Equipment (NOCSAE) headform, the study also emphasises the importance of evaluating several impact locations. However, the STAR assessment was limited to MIPS and conventional helmets. Here, we expanded on these results with a wider range of helmet technologies, including WaveCel, SPIN and an airbag helmet. Moreover, we determined the influence of these technologies on the brain using our computational model of TBI.

The airbag helmet, Hövding, outperformed all helmets by far in most metrics except PRV and BrIC when considering a 75 ms duration. When considering only the first 30 ms of impact, the airbag helmet outperforms all helmets across all injury metrics considered in this study. Reasons for this are likely due to the impact kinematics which result from the large size and low stiffness of the helmet. These features result in a prolonged impact period (~3 times longer than conventional) with a significantly lower peak acceleration as seen in Fig. 3b and shown in previous work.^32^ Analysis of the high-speed videos reinforces this, revealing that the headform has rotated noticeably less than conventional helmets during the same time period. Our brain model shows that this is favourable with respect to brain tissue strain. However, the prolonged duration of impacts with this helmet means that the effect of the neck is likely to be considerable in a real-life impact. Furthermore, due to the size of the airbag, interaction with the shoulder and neck during the impact is likely. It is also noteworthy that BrIC was developed on the basis of 30 ms impacts,^50^ and hence may not be suitable for longer duration impacts such as with the Hövding or similar future technologies.

We determined strain in the corpus callosum and sulci during impacts. Corpus callosum is the largest white matter tract in the human brain and a common location of axonal injury after severe TBI.^47^ Previous work has shown clear relationship between mechanical strain and pathology, including axonal damage and neuroinflammation.^4^,^18^ These pathologies can persist several years after an injury and have been shown to contribute to accumulation of tau proteins in depths of sulci in cases of chronic traumatic encephalopathy.^11^ In an in-vivo experiment on guinea pig’s optic nerve, Bain et al. determined a 0.21 strain threshold for producing structural damage.^53^ A recent study using an in-vivo controlled cortical impact model in rats has shown that increasing strain from below 0.1 to around 0.4 increases axonal damage and neuroinflammatory responses in white matter.^6^ This suggests that decreasing strain is an effective way of reducing pathology. Hence, we predicted strain in corpus callosum and sulci to, for the first time, determine the effects of the new helmet technologies on reducing strains in these key regions of the brain. The predicted strains were in the range reported in this recent animal work, though due to differences in biology and computational models, we cannot directly compare the results. However, this previous work again confirms that the new technologies, which reduce the strain in the brain and key anatomical regions are effective methods for reducing axonal damage and neuroinfalmmation post-injury.

This study has some limitations. We used a Hybrid III headform in this study, which is one of the most biofidelic headforms with regards to the head shape and size, mass and moments of inertia.^28^ However, it has a vinyl rubber skin which has a larger coefficient of friction in contact with fabric than the human scalp.^52^ The coefficient of friction of the surrogate skin should be improved in future to produce more biofidelic test conditions. Similar to previous studies,^14^ we used an isolated headform, thus ignored the potential effects of the neck during impacts. Several studies have shown that primary peak loads from head-first impacts are less affected by the presence of a neck.^40^,^44^ Some studies on helmets have used a HIII neck, but this neck has limitations, such as stiffness in axial loading,^8^ which can have adverse effects on the results.^45^,^55^ Future work should address the development of a surrogate neck that is biofidelic in head-first impacts. This should enable current and future helmets, particularly those that produce head accelerations with longer durations than conventional helmets, such as the airbag helmet, to be evaluated with improved fidelity. Finally, we have attempted to assess similar helmets with and without the technologies where possible (i.e. Giro Caden MIPS vs no MIPS, Biltema MIPS vs no MIPS). However, we were limited with the availability of helmets with and without the same technologies in the current market. Notably, the Biltema helmet versions in this study have other design differences not exclusive to MIPS that may have contributed to some of the performance differences seen between the helmet versions.

A final notable limitation is regarding the statistical evaluation method employed. We used a z-scoring approach to test whether an individual helmet performed better or worse than ‘conventional helmets’. This method enables future comparisons of new helmets to be tested against an established control or benchmark group of helmets. However, we are limited in this study by a small control sample size, which can lead to potential biases. This method can be optimised in future by testing the ‘diagnostic accuracy’ in the context of a different control sample sizes.

In summary, our assessment of 27 commercially available bicycle helmets shows that the majority of helmets with new technologies have the potential to reduce peak rotational acceleration and velocity and maximal strain in corpus callosum and sulci in oblique impacts. However, the outcome is highly sensitive to impact location. Hence, incorporating different impact locations in future oblique impact test methods and designing helmet technologies for the mitigation of head rotation in different planes are key to reducing brain injuries in bicycle accidents, where helmets are worn.

## Appendix

### Kinematic-based metrics

See Appendix Tables 2, 3, 4, 5.

### Brain strain-based metrics

See Appendix Tables 6, 7, 8.
